# Annotations

**Published:** 1900-09-29

**Authors:** 


### Annotations.

### The Pharmaceutical Society.

We hear that a curious tempest in a teapot has arisen in the Pharmaceutical Society, in consequence of a dispute between the London Council and the Examining Board of the Scottish branch as to the fee which should be charged to a candidate who, having paid for his examination and passed the practical portion of it, is prevented by illness or other valid cause from attending for the second or oral portion at the appointed time. The Scottish Board maintain that a merely nominal fee should be charged for the second portion when the candidate is able to present himself; the London Council maintain that the second portion should be paid for over again. The amount at issue is not large, and cannot exceed three guineas; while the principle may evidently be regarded from different points of view. It may reasonably be held that the candidate has paid for the whole examination, and should not be called upon to pay again for any part of it. It may also be held that the society has earned its money by providing a Board of Examiners at the appointed time, and that the candidate may justly suffer for his own inability to take advantage of the provision. So far, it is a question the like of which often comes before the law courts, the question which, of two innocent parties, should bear the consequences of an occurrence for which neither can be blamed. We cannot but think that it would be an error of judgment to afford increased facilities for the practice of dividing an examination which ought to be one and indivisible. The candidates ought to be prepared for the whole at once, and to permit the frequent postponement of the second part would be to give opportunity for cramming of a highly objectionable kind. Where the illness is real, or the obstacle valid, the candidate may be sympathised with for the loss of the money which he has paid; but, at the same time, it does not seem impossible that, if the practice of charging a nominal fee for the deferred second part were once recognised and adopted, the strain thrown by the first part upon the health of the candidates might be found, in future, to be more serious than it has been in the past.

### A Hardy Old Doctor.

A few weeks ago a hardy old doctor in his seventy-eighth year, Mr. C. J. Harris, of Kilburn Priory, wrote to the *Lancet* saying that for more than four years he had been a total abstainer from meat, alcohol, and tobacco, and that he had just returned from riding to Edinburgh and back on his tricycle. He was anxious to know whether anyone else of his age had accomplished a similar journey either on flesh or vegetarian diet. He was 31 days away from home, his weight on his return was the same as when he started, namely, 116 lb. net, his tricycle weighed 45 lb., and his luggage about 60 lb. It was geared up to 72, and his cranks were seven inches in length. We certainly may congratulate him on his well-maintained strength and suppleness of limb. Further details of his performance, which have since been given, are not without interest. He did not eat flesh food in any form nor even eggs, nor did he touch alcohol or tobacco. His food seems to have been wholemeal bread and biscuits, a little cheese, tomatos, bananas, chocolate, butter, marmalade, and jam, sometimes vegetables or pudding, a few times porridge, and once some bread and milk. He did not eat at stated times, but (his teeth having all disappeared) would munch some bread or biscuit with some sweets or chocolate as he rode along. He took cocoa and milk at breakfast and at his evening meal, otherwise he did not drink much during the day, but he drank water sometimes pretty freely during the night. Altogether it was an interesting performance, and it shows well enough what he wished it to show, that a man who can digest his food well can get plenty of energy out of what to some would seem a self-denying diet. But it was a good diet of a fairly varied nature, and no doubt it contained quite as much proteid as was required. The great point, of course, was that it suited his digestion. As to the other question, whether a lifelong abstinence from meat and alcohol and tobacco would have produced so hale and hearty an old man, of course the experiment tells nothing.

### Waists and Corsets.

Attention has been lately drawn by certain medical men to the disproportion which exists between the measurement of the female waist taken outside the corset and that taken on the nude figure. We should not have thought that there was much novelty in the discovery that the nude measurement is the bigger of the two. For years, nay for centuries, the complaint has gone up that women persist in compressing the waist under the idea that this proceeding adds to their beauty. It must be confessed also that many of them, and those not always the silly ones, maintain that it also adds to their comfort. This we greatly doubt, although it is not improbable that a lady who has accustomed herself to the spurious support these instruments give may for a time feel somewhat lost without some compression. One writer, who has measured many scores of young women, says that they mostly measure about two inches less outside the corset than around the corresponding part of the nude waist, while another, who only confesses to 50 such measurements, gives the actual averages of his observations, which go to show that the average female waist in his part of the world measures 23·8 inches, while the average corset, by which these waists are covered, is but 21·7 inches. Let the young ladies who are anxious about their "figures" ponder over these figures also, and give a thought to their much-compressed and long-suffering insides. We admit the beauty of a neat figure and the attractiveness of a small waist. Nay, we will go further, and confess that many of the most active and healthy young women have what we should call small waists, although they probably would not be so designated by a fashionable corsetière. But a waist that is made small by aid of corsets is another matter, and those who have had occasion to observe the stains and creasings of the skin, the displaced liver, and, in later life, the protruding, pendulous abdomen hanging down over the thighs, which the production of a small waist so often involves, will hesitate, we think, to call it a thing of beauty. A neat figure is chiefly a matter of heredity and original build. But those on the borderland, who feel that their inches are becoming a somewhat anxious question, should seek beauty in diet, exercise, and *régime*, certainly not in corsets and compression.

